# Gut Microbiome as a Source of Probiotic Drugs for Parkinson’s Disease

**DOI:** 10.3390/ijms26199290

**Published:** 2025-09-23

**Authors:** Elena U Poluektova, Alla Stavrovskaya, Anastasia Pavlova, Roman Yunes, Maria Marsova, Tatiana Koshenko, Sergey Illarioshkin, Valery Danilenko

**Affiliations:** 1 Vavilov Institute of General Genetics, Russian Academy of Sciences, Moscow 119991, Russia; masha_marsova@mail.ru (M.M.); tatiana_koshenko@mail.ru (T.K.); valerid@vigg.ru (V.D.); 2 Russian Center of Neurology and Neurosciences, Moscow 125367, Russia; alla_stav@mail.ru (A.S.); pav_nastasya@mail.ru (A.P.); snillario@gmail.com (S.I.)

**Keywords:** Parkinson’s disease, microbiota, probiotics, postbiotics, extracellular vesicles, live biotherapeutic products

## Abstract

Parkinson’s disease (PD) is a progressive, irreversible neurodegenerative disorder characterized by motor impairments and a wide spectrum of non-motor symptoms, including gastrointestinal dysfunction, sleep disturbances, depression, and cognitive decline. These manifestations arise from disturbances across multiple systems—gastrointestinal, neuroendocrine, immune, enteric, and central nervous systems. Alterations in the gut microbiota may play a causal role in PD onset and frequently accompany disease progression. The gut–brain axis, particularly the vagus nerve, is increasingly recognized as a key communication pathway whose dysregulation contributes to systemic dysfunction and the breakdown of homeostasis, ultimately driving PD pathology. Currently, there is no cure for PD, and existing treatments primarily target symptom relief. Effective management of PD requires a comprehensive approach that integrates multiple pharmacologically active agents aimed at restoring impaired organ functions and, when possible, neutralizing toxic factors that accelerate disease progression. One promising therapeutic avenue lies in functional gut bacteria, which form the basis for developing live biotherapeutic products, postbiotics, and bacterial vesicles. In this review, we summarize current data on the effects of probiotics in PD, drawing on both animal models and clinical studies. We highlight the role of probiotics in modulating PD pathophysiology and discuss their potential as adjunctive therapeutic agents. To provide a broader perspective, we also include sections describing the clinical manifestations of PD, gut microbiota alterations associated with the disease, and the role of artificial intelligence, particularly machine learning, in constructing functional models of PD.

## 1. Introduction

Parkinson’s disease (PD) is a progressive, irreversible neurodegenerative disorder that affects multiple organ systems, including the gastrointestinal, neuroendocrine, immune, enteric, and central nervous systems (CNS) [1,2]. A key biomarker of PD is the formation of Lewy bodies in the neurons of the substantia nigra, resulting from the misfolding and aggregation of the alpha-synuclein protein [3]. The gut–brain axis is considered one of the body’s most important communication systems [4,5]. According to the Braak hypothesis, the initial trigger of PD may be an unknown pathogenic factor in the gut [6]. This factor could induce alpha-synuclein misfolding in enteric neurons, followed by the translocation of misfolded proteins and their mediators into the CNS [7]. In recent years, gut microbiota bacteria have been investigated as potential mediators of alpha-synuclein misfolding [8], and neuronal and behavioral dysfunctions [9,10] and prion-like mechanisms have also been proposed [11]. Strategies to prevent this process include immunotherapeutic approaches such as active immunization with peptide vaccines designed to elicit an immune response against insoluble or oligomeric alpha-synuclein and passive immunization with antibodies that directly target alpha-synuclein aggregates (e.g., Prasinezumab, Cinpanemab) [12].

However, current findings suggest the existence of additional mechanisms driving protein misfolding and transfer. It is now established that bacterial vesicles from the gut microbiota and human exosomes can package and transport proteins, metabolites, small RNAs, and other signaling molecules across the intestinal and blood–brain barriers [13,14,15]. This emerging knowledge highlights new avenues for identifying therapeutic targets in PD. In recent years, increasing attention has been directed toward the therapeutic potential of probiotic strains. Several probiotics and their formulations are currently in clinical trials as candidate treatments for PD.

In this review, we present an overview of current findings on the effects of probiotics in PD, based on evidence from animal models and clinical studies, with the aim of clarifying mechanisms of bacterial action and evaluating their therapeutic potential. To provide better context, we also include sections summarizing the pathology of PD, characteristic alterations in the gut microbiota, and the contribution of artificial intelligence, particularly machine learning, in developing functional PD models. We critically analyze recent experimental studies in this field, with a focus on the development of therapeutic products derived from functional bacteria with neuromodulatory, immunomodulatory, and anti-inflammatory (including neuroinflammatory) properties.

## 2. Current Understanding of the Development of Parkinson’s Disease

### 2.1. Etiology of Parkinson’s Disease

PD is a slowly progressing chronic neurodegenerative disorder that affects 1–4% of individuals over the age of 60. It occurs in both sporadic and hereditary forms, with contributing factors including genetic mutations and environmental exposures. PD is classified into primary (idiopathic), secondary (symptomatic), and neurodegenerative PD subtypes [16,17]. A hallmark of PD is the degeneration of neurons in the substantia nigra pars compacta (SN) and the accumulation of α-synuclein–containing protein aggregates (Lewy bodies). Damage to the nigrostriatal pathway leads to dopamine depletion in the striatum, which underlies the characteristic motor symptoms of the disease, such as hypokinesia, rigidity, and resting tremor. Involvement of additional neuronal pathways contributes to cognitive and autonomic dysfunctions [18,19].

Peripheral nervous system involvement has also been reported, including α-synuclein accumulation in the enteric nervous plexuses and salivary glands, which is associated with gastrointestinal dysmotility and impaired salivation [20]. Both exogenous (environmental) and endogenous (genetic) factors are implicated in PD pathogenesis. Endogenous factors include Mendelian-inherited genetic mutations. Monogenic forms of PD are linked to six of the 28 chromosomal regions associated with the disease. However, incomplete penetrance, variable expression, and phenocopies complicate assessment of whether PD is exclusively genetic or influenced by environmental modifiers [21].

Environmental exposure, particularly to pesticides and industrial chemicals, is associated with increased PD risk. Chronic exposure to rotenone, paraquat, and solvents such as trichloroethylene and perchloroethylene in humans has been shown to promote Lewy body formation, α-synuclein misfolding, and mitochondrial dysfunction, ultimately triggering oxidative stress and apoptosis. Irreversible parkinsonism can also be induced by neurotoxins such as 6-hydroxydopamine (6-OHDA) and 1-methyl-4-phenyl-1,2,3,6-tetrahydropyridine (MPTP), which are metabolized into toxic compounds that accumulate in dopaminergic neurons and lead to their degeneration. Traumatic brain injury is another contributing factor, as it can damage the basal ganglia and their connections, disrupt the blood–brain barrier, and initiate prolonged neuroinflammation, mitochondrial dysfunction, excessive glutamate release, and α-synuclein aggregation in the brain. Together, these mechanisms may contribute to the development of secondary parkinsonism [17,22].

### 2.2. Motor and Non-Motor Symptoms in Parkinson’s Disease

Parkinson’s disease manifests through motor disturbances such as hypokinesia, muscle rigidity, resting tremor, and postural instability. The characteristic “petitioner’s posture” in PD is associated with increased tone in the flexor musculature [23,24]. Disease progression involves not only the dopaminergic system but also the cholinergic, serotonergic, and noradrenergic systems of the brain. Damage to these systems contributes to the development of non-motor symptoms (NMS), which persist throughout the disease course.

NMS are classified as autonomic, sensory, sleep-related, and neuropsychiatric. Autonomic dysfunction can affect nearly all functional systems of the body. Among the most common are gastrointestinal disturbances, which often appear in the early stages of PD—sometimes even before motor symptoms. These include impaired intestinal motility, dysphagia (difficulty swallowing), and gastroparesis (delayed gastric emptying), which hinder the absorption of medications, particularly levodopa, thereby reducing therapeutic efficacy. Patients may also experience heartburn, reflux, and other digestive problems caused by altered gastric and sphincter motility [20,25].

Olfactory dysfunction (hyposmia) is another frequent early marker of PD [26]. The disease also disrupts the regulation of sleep and wakefulness, leading to insomnia, excessive daytime sleepiness, and impaired motor control during REM sleep [27]. Cognitive impairments and dementia typically emerge in later stages, manifesting as reduced attention, memory deficits, and impaired visuospatial orientation. Common psychiatric disturbances include depression, apathy, hallucinations, and impulse control disorders, the latter often induced by pharmacological therapy [28]. Some NMS may also arise directly as side effects of treatment.

Despite their key role in PD, NMS remain underdiagnosed and insufficiently studied.

### 2.3. Pathogenesis of Parkinson’s Disease

Catecholamines, including dopamine, are synthesized from L-tyrosine through the action of tyrosine hydroxylase (TH), the rate-limiting enzyme in dopamine biosynthesis. In patients with PD, TH activity is significantly reduced in the nigrostriatal system, as well as in other brain regions, reflecting the systemic nature of neurodegeneration [29,30]. Dopamine metabolism generates reactive oxygen species (ROS) and quinones, which further exacerbate mitochondrial dysfunction and promote aggregation of α-synuclein—a protein central to PD pathogenesis [31,32].

Morphologically, PD is characterized by Lewy bodies—intracellular aggregates of abnormal, insoluble α-synuclein—found in both central and peripheral nervous system structures. Their widespread presence, particularly in advanced stages, confirms the progressive nature of the disease. The extent of dopaminergic cell loss correlates with Lewy body burden [33,34].

A hallmark of PD is oxidative stress, driven by mitochondrial dysfunction and ROS accumulation. Dopaminergic neurons are especially vulnerable due to their high oxygen metabolism, low antioxidant capacity, and elevated iron content. Increasing evidence highlights oxidative stress as a major driver of dopaminergic neurodegeneration across all forms of PD [35,36].

Iron plays a central role in free radical–producing oxidative reactions. Under physiological conditions, the SN is among the brain regions with the highest iron content. Abnormally elevated iron levels in the SN have been demonstrated in both genetic and idiopathic PD, linking the disease to disrupted iron metabolism [37]. With aging, glutathione levels and other antioxidant defense components decline, rendering neurons less capable of mounting stress responses [38].

Chronic neuroinflammation is another key factor in PD pathogenesis. Damaged neurons release α-synuclein, which activates astrocytes and microglia. These glial cells, in turn, produce proinflammatory cytokines, chemokines, and ROS, further amplifying tissue injury and neurodegeneration [39,40,41].

Taken together, these findings support the view of PD as a multifactorial disorder driven by diverse pathogenic processes at the cellular level. The human microbiota—particularly the gut microbiota—also contributes to PD onset and progression. Alterations in gut microbial composition may influence disease mechanisms, exacerbate inflammation, and aggravate symptoms. Moreover, the microbiota is increasingly studied as a potential source of therapeutic agents or adjuvants in PD treatment. These aspects will be discussed in the following sections of this review.

### 2.4. Modeling Parkinson’s Disease in Rodents

Postmortem analysis of PD-affected human brain tissue provides insights primarily into the terminal stages of the disease and is unsuitable for investigating early neurodegenerative events. Therefore, experimental model systems are essential for studying different aspects of PD. Commonly used models include rodents (rats and mice), non-human primates, and model organisms such as *Caenorhabditis elegans*, *Drosophila melanogaster*, and zebrafish. Among these, rodents are the most widely used and accessible. To reproduce disease symptoms, researchers employ both genetic models (e.g., knockout mice for genes such as α-synuclein, Parkin, UCH-L1, PINK1, DJ1, LRRK2, or overexpression of mutant human α-synuclein) and neurotoxic models [42]. Exogenous neurotoxins remain the most frequently applied approach [43].

The first PD animal model was established through intracerebral injection of 6-OHDA in rats [44]. 6-OHDA is a structural analog of catecholamines (dopamine and norepinephrine) that selectively destroys dopaminergic neurons by generating ROS. Since it cannot cross the blood–brain barrier (BBB), stereotactic surgery is required to deliver it directly into the SN [45,46]. A more widely used model involves MPTP, which readily crosses the BBB and therefore does not require surgical administration. MPTP is metabolized into the toxic compound MPP^+^, which damages dopaminergic neurons and replicates both motor and non-motor symptoms of PD [47]. MPTP administration in monkeys is considered the gold standard, though similar studies are also conducted in rodents [48].

Agricultural pesticides such as paraquat and rotenone also target the dopaminergic system. Paraquat, a nitrogen-based herbicide widely used to control broadleaf weeds, is structurally similar to MPP^+^, the toxic MPTP metabolite. Rotenone, used to eliminate invasive fish in lakes and reservoirs, is a potent dopaminergic neurotoxin with high lipophilicity that allows it to cross the BBB and enter cells. However, its application is limited by systemic toxicity. Both paraquat and rotenone disrupt mitochondrial function, increase ROS production, and lead to dopaminergic neuron loss and motor deficits [49,50,51].

## 3. Gut Microbiome and Parkinson’s Disease

**Figure 1:**
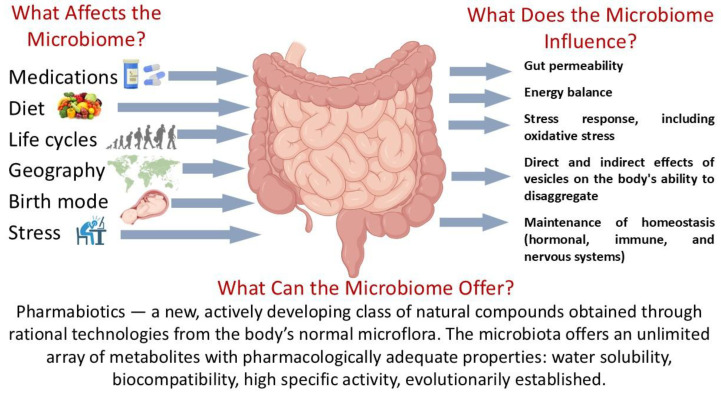
The human gut microbiome: functional activity in the norm. Parts of this figure were created in BioRender (2025) https://BioRender.com/135e66q (accessed on 1 July 2025).

The study of the human gut microbiome has become one of the most rapidly advancing interdisciplinary fields in biomedical science. A central objective—identifying key parameters and characterizing the microbiome in its healthy state—has been set and is being addressed with increasing success [52,53,54]. Over the past decade, the idea has emerged that the microbiome functions as an independent organ, integrating environmental factors with major physiological systems of the human body through the ENS and CNS via the gut–brain axis [55] (Figure 1).

The relationship between gut microbiota and PD is complex. The microbiome may serve as a reservoir, a trigger, and even a source of therapeutic agents for PD. The Braak hypothesis and its supporting evidence were discussed in the Introduction. Additional confirmation that gut microbiota can act as a disease trigger comes from studies showing that fecal transplantation from PD donors to recipient mice induces not only disruptions of gut homeostasis but also motor and pathological features characteristic of PD [56].

Further studies have distinguished gut-first and brain-first PD subtypes. In 2019, Borghammer and Van Den Berge demonstrated different distributions of alpha-synuclein aggregates in postmortem PD patients: in one group, aggregates were predominantly located in the brain (brain-first), while in the other, they were mainly in the gut (gut-first) [57]. This led to the hypothesis that PD pathology originates either in the gut or in the brain. Gut-first PD is characterized by symmetric motor symptoms and bilateral brain involvement, whereas brain-first PD typically begins in one hemisphere, producing asymmetric motor symptoms [58]. Differences in the severity of gut microbiota alterations among PD patients are likely attributable to subtype. Indeed, the most pronounced microbiota changes are observed in patients with symmetric motor symptoms [59].

**Figure 2:**
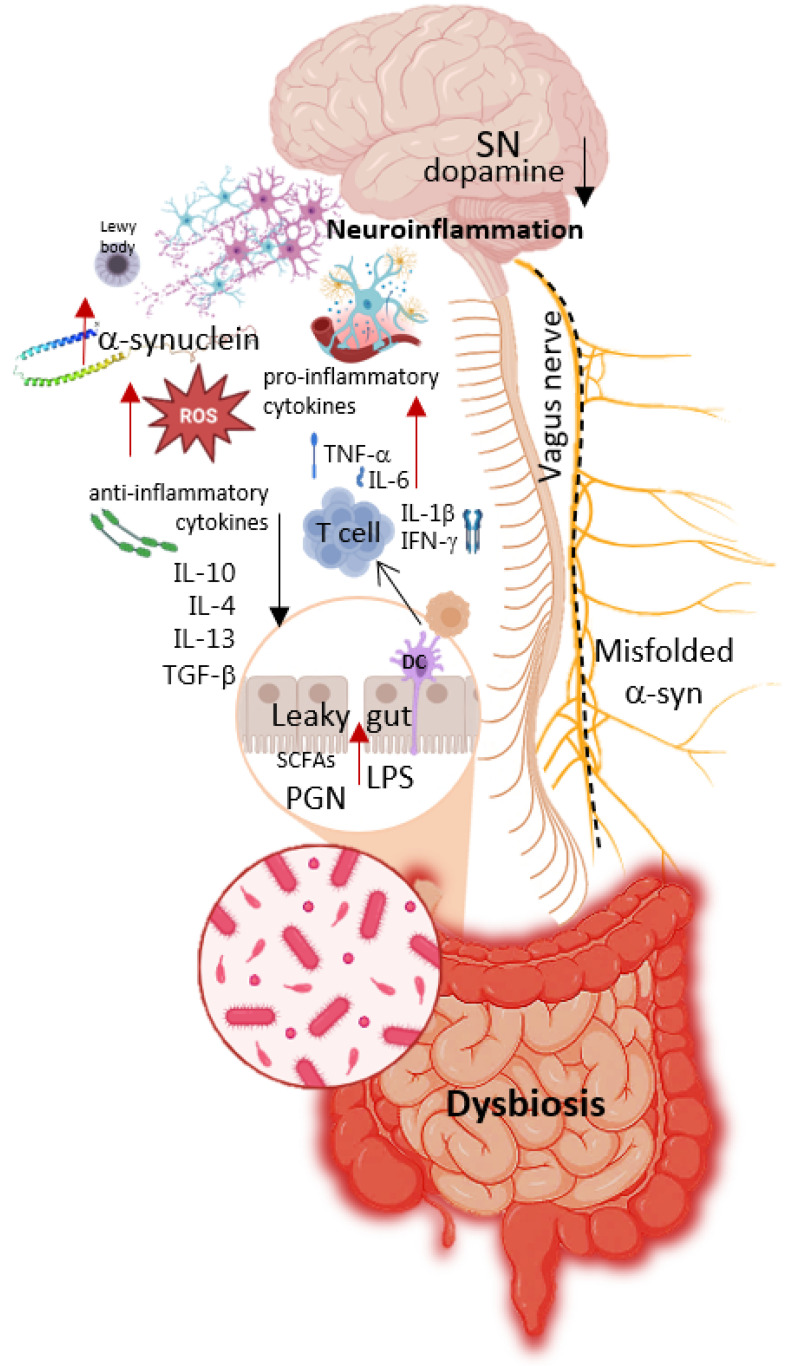
Relationship between negative changes in microbiome composition (dysbiosis) and subsequent stages of Parkinson’s Disease development. Parts of this figure were created in BioRender (2025) https://BioRender.com/8bysho5 (accessed on 1 July 2025).

A significant portion of patients with PD exhibit gut dysfunction, including inflammatory processes, increased intestinal permeability, and dysbiosis [60]. Figure 2 schematically illustrates the relationship between negative changes in microbiome composition (dysbiosis) and the subsequent stages in the development of PD. Key mechanisms involved include oxidative stress and changes in the enteric nervous system, which, through neuroinflammation, lead to aggregation of α-synuclein proteins and the formation of Lewy bodies [61]. Simultaneously, there is immune system dysregulation, resulting in persistently elevated levels of pro-inflammatory cytokines, as well as dysfunction of the vagus nerve, disrupting communication between the enteric nervous system and the brain [7]. At later stages, these processes culminate in neurodegeneration, particularly the degeneration of dopaminergic neurons and a decline in dopamine levels.

PD is also associated with alterations in the structure and metabolic activity of the microbiota. Dysbiosis is common, though not universal—approximately half of patients show no microbial imbalance. Compared with healthy controls, PD patients exhibit differences in beta [62] and alpha [63] diversity, as well as shifts in taxonomic composition, though results vary across studies. At the genus level, the most frequent changes include increases in *Christensenella*, *Oscillospira*, *Clostridium*, *Enterococcus*, *Streptococcus*, *Lactobacillus*, *Alistipes*, *Parabacteroides*, *Bifidobacterium*, *Escherichia/Shigella*, *Bilophila*, *Desulfovibrio*, and *Akkermansia*, along with decreases in *Blautia*, *Roseburia*, *Fusicatenibacter*, *Faecalibacterium*, *Streptococcus*, *Prevotella*, and *Haemophilus* [62]. Many studies consistently report reductions in butyrate-producing genera such as *Faecalibacterium* and *Roseburia*, together with increased abundances of *Akkermansia* and *Bilophila*, as microbial signatures of PD [64]. Although *Bifidobacterium*, *Lactobacillus*, and *Akkermansia* are generally considered beneficial, their elevated levels in PD may reflect confounding factors: *Bifidobacterium* and *Lactobacillus* increases are often associated with PD medications, particularly COMT (catechol-O-methyltransferase) inhibitors, while higher *Akkermansia* levels may relate to aging [62]. Importantly, *Akkermansia muciniphila* can degrade the intestinal mucus layer, thereby increasing gut permeability and potentially driving inflammation and oxidative stress [65]. Identifying microbiota changes directly linked to PD remains challenging. More robust insights can be obtained through statistical analyses of bacterial groups. For instance, analysis of 35 bacterial groups distinguished individuals with and without PD with an accuracy of just over 84% [64]. The most effective machine-learning approaches for such microbiome analyses are described in Section 5.

At the species level, most studies report that *Blautia wexlerae*, *Faecalibacterium prausnitzii*, and *Roseburia* are depleted in PD patients, whereas *Clostridium leptum*, *Bifidobacterium bifidum*, *Bifidobacterium dentium*, *Eisenbergiella tayi*, *Lactobacillus salivarius*, and *A. muciniphila* are increased. Another noteworthy finding is the higher prevalence of the poorly studied species *Ruthenibacterium lactatiformans* in PD. This species shares a high degree of genetic similarity with *F. prausnitzii*, and competition between the two may underlie the observed PD associations [66,67].

Beyond taxonomic composition, the metabolic activity of gut microbiota is of critical importance. PD is associated with a more pro-inflammatory microbiome. The phylum *Proteobacteria*, which contains many pro-inflammatory taxa, is frequently overabundant in PD [62]. The pro-inflammatory effects are largely attributed to lipopolysaccharides (LPS)—endotoxins that form part of the outer membrane of Gram-negative bacteria [68]. LPS activate TLR4 signaling, leading to increased cytokine production, oxidative stress, and microglial activation. According to the endotoxin hypothesis of PD, gut dysfunction elevates LPS levels in both the gut and bloodstream, thereby promoting α-synuclein aggregation in enteric neurons and triggering peripheral—and subsequently systemic—inflammation [69].

Pro-inflammatory activity may also result from reduced short-chain fatty acids (SCFAs). PD patients often exhibit decreased fecal SCFA levels [63], but elevated plasma SCFAs, with these alterations correlating with disease severity [70]. Collectively, these findings suggest that SCFAs are implicated in both the onset and progression of PD [71]. SCFAs—primarily acetic, propionic, and butyric acids—are produced by bacterial anaerobic fermentation of dietary fiber in the gut. They represent the main energy source for colonocytes, can cross the blood–brain barrier, possess anti-inflammatory properties, and play a direct or indirect role in microbiota–gut–brain axis signaling [72,73]. Many SCFA-producing bacteria (*Blautia*, *Roseburia*, *Faecalibacterium*, *Eubacterium*, and *Prevotella*) are consistently depleted in PD, whereas others (*Bifidobacterium* and *Akkermansia*) are increased [62,74]. Under certain conditions, however, SCFAs may also modulate signaling cascades that exacerbate PD pathology [71,75]. A comprehensive evaluation of SCFA effects is therefore needed—one that accounts for the diversity of SCFA types, their concentrations, and the host cellular environment—together with an assessment of the “optimal range” of SCFA-producing bacteria.

PD-associated microbes also perform protein fermentation, generating toxic metabolites. One such metabolite is p-cresol, a unique bacterial product not synthesized by human enzymes. Derived from tyrosine and phenylalanine catabolism, p-cresol promotes reactive oxygen species (ROS) production, thereby driving inflammation and multiple physiological abnormalities [76]. Elevated p-cresol metabolites have been detected in the serum of PD patients [77]. Their production has been linked to *Blautia obeum*, a bacterium associated with PD [59].

Five of the six major categories of carbohydrate-active enzymes (CAZymes) were decreased in PD. Pathway analysis further revealed a marked reduction in genes involved in riboflavin and biotin biosynthesis. Metabolomic profiling of fecal samples also demonstrated significantly lower polyamine levels in PD patients [63].

When assessing microbiota composition in PD, it is essential to account for the fact that patients are invariably receiving medication. Many drugs, including non-antibiotics, can alter the gut microbiota by modifying its composition and activity. In turn, gut microbes may influence drug bioavailability, bioactivity, or toxicity [78]. Levodopa itself appears to exert only minor effects on microbiota composition [79]. By contrast, COMT inhibitors—administered alongside levodopa and carbidopa to prevent levodopa degradation—have a more pronounced impact. Entacapone, a COMT inhibitor, alters microbial activity by chelating and depleting available iron, thereby reshaping gut microbial composition and function [80]. Conversely, certain gut bacteria (*Enterococcus* and *Bifidobacterium*) can metabolize levodopa through deamination or decarboxylation, reducing its bioavailability [81,82].

PD patients also frequently present with altered oral health [83] and oral dysbiosis [84], suggesting that oral inflammation may contribute to disease development.

The gut microbiome is increasingly recognized not only as a therapeutic target but also as a source of novel therapeutic agents. The most promising candidates are functional bacteria present in the microbiomes of healthy individuals. Computational algorithms for the discovery of such agents have already been applied to various psychotropic compounds [5,53,85]. The first step in this strategy involves a comprehensive analysis of published datasets to define a reliable reference set of bacterial genes and create catalogs. These catalogs enable the characterization of specific microbiome functions—such as neuromodulatory, immunomodulatory, or antioxidant activities—and facilitate the identification of relevant genes and metabolites.

One promising approach is the use of metagenomic signatures: matrices that include information not only on genes but also on the bacterial species harboring them. This strategy allows researchers to identify and isolate specific strains from the human gut metagenome and characterize them through multi-omics technologies. Genomic, transcriptomic, proteomic, and metabolomic profiling can then be applied to select strains with desirable functional properties from existing probiotic collections. Such strains may subsequently be developed into live biotherapeutic products or pharmabiotics. The ultimate goal is to generate pharmabiotics with defined pharmacological activities that restore gut function, repair damage to other organs and systems (Figure 2), and alleviate Parkinsonian symptoms [86,87].

## 4. Probiotics and Metabiotics: Existing Potential for the Development of Live Biotherapeutic Products and Pharmabiotics

### 4.1. Probiotics in Parkinson’s Disease: Analysis of Neuroinflammatory, Immunomodulatory, and Neuromodulatory Activity in Animal Studies

Symbiotic bacteria from the human gut microbiota are widely used to alleviate symptoms and treat various human diseases [88]. A substantial number of studies have explored the potential use of these bacteria as adjuvants in PD therapy. A search in PubMed NCBI using the query “probiotics Parkinson Parkinson’s” returns more than 300 publications from 2020 to April 2025. Most experimental studies have been conducted in animal models, primarily rodents. The findings of many of these studies have been summarized in reviews [89,90,91,92,93,94]. This article focuses primarily on experimental studies conducted between 2024 and April 2025, as well as earlier investigations employing alternative animal models. Regarding clinical studies on probiotic interventions in PD patients, we aimed to include all available reports. Relevant articles were identified through searches in Google Scholar and PubMed using the keywords “probiotic(s) Parkinson’s (Parkinson),” “Bifidobacterium Parkinson’s (Parkinson),” and “Lactobacillus Parkinson’s (Parkinson).” Studies were then selected according to two criteria of interest: (i) probiotic use in animal models of PD, and (ii) probiotic interventions in clinical trials. The overarching goal was to evaluate the mechanisms of bacterial action and assess their potential for clinical application.

Table 1 provides a list and a brief summary of studies from 2024–2025 using bacterial preparations in animal models of PD. Various bacterial species were tested: *Lactocaseibacillus* (*Lactobacillus*) *rhamnosus*, *Lactiplantibacillus (Lactobacillus*) *plantarum*, *Limosilactobacillus* (*Lactobacillus*) *fermentum*, *Limosilactobacillus* (*Lactobacillus*) *reuteri*, *Lactobacillus delbrueckii*, *Lactobacillus acidophilus*, *Ligilactobacillus* (*Lactobacillus*) *murinus*, *Bifidobacterium animalis* subsp. *lactis*, *B. bifidum*, *Bifidobacterium longum*, *Bifidobacterium breve*, *Lactococcus lactis*, *Escherichia coli*, *Streptococcus thermophilus*, *Bacillus subtilis*, *Bacillus coagulans*, *Clostridium butyricum*, and *A. muciniphila*. Many of these bacteria have been well-characterized in other models (e.g., *L. rhamnosus* GG, *L. plantarum* PS128, *A. muciniphila* Akk11). Both individual strains and multi-component mixtures, as well as pharmaceutical formulations (e.g., VSL#3^®^, a probiotic used for IBS treatment), were tested.

The biological effects of probiotic strains are generally species- and strain-specific. This is clearly demonstrated in cell culture experiments. For example, 48 bifidobacterial strains were tested on an LPS-induced BV-2 murine microglial cell line (a neuroinflammation model) for NO signaling, Nrf2/HO-1/NQO1 pathway gene expression, and cytokine production. Only one strain, *B. longum* subsp. *longum* C7, showed promising protective properties [107]. Testing many strains in animal models is more complex, but strain specificity was also observed in such models [109]. In some cases, probiotic effects were found to be species-specific rather than strain-specific [99]. Genetically engineered strains were also used in some studies [110,111]

The administered bacterial doses ranged from 10^8^ to 10^10^ CFU, and it was observed that higher doses yielded stronger effects [96,104]. The duration of experiments varied from 2 to 8 weeks. Prolonged administration was considered most effective [112].

Some studies combined bacterial preparations with medications or dietary supplements. *L. rhamnosus* increased the bioavailability of curcumin [96]. Vinpocetine (a synthetic alkaloid believed to improve cerebral circulation) showed effects similar to the bacterial preparation [97]. Liraglutide (a GLP-1R agonist that protects dopaminergic neurons by reducing inflammation) exhibited neuroprotective and anti-inflammatory effects and regulated gut microbiota dysbiosis, like *L. plantarum*, but did not affect intestinal transit, unlike the bacterial preparation [101]. The effects of *B. longum* on several markers were comparable to L-DOPA, and it outperformed L-DOPA in reducing inflammation [107]. When a bacterial mixture was co-administered with L-DOPA–benserazide, the neuroprotective effects of the bacteria did not interfere with L-DOPA therapy; in fact, the pharmacological efficacy of L-DOPA was maintained and, in some cases, improved [103]. Thus, in all studies, bacterial treatments either mirrored or enhanced the effects of conventional drugs.

In addition to variations in bacterial formulations, experimental designs also differed. Some studies used an acute model with a single high dose of a neurotoxin [95,98], while others employed a chronic model with repeated low doses [96,97,103]. Bacterial preparations were administered before the neurotoxin [101,102,105,107], after the neurotoxin [95,100,104], or simultaneously [96,97,98,103]. The experimental design most closely resembling human disease progression was employed by [100], in which 6-OHDA was injected into the SN, followed by a one-month latency period until PD symptoms emerged. Only then were bacterial therapies initiated. Animals used were generally 8–10-week-old mice, though [103] used 1-year-old mice, making the model more representative of the human disease. Most studies used male animals, with female subjects rarely included [104]. When both sexes were used, sex-specific differences in probiotic effects were observed. For instance, *L. plantarum* improved memory and reduced TNF-α levels in female mice, but these benefits were not seen in males [113].

All the reviewed studies clearly demonstrate the undeniable effect of bacterial preparations in alleviating PD symptoms. However, it is not possible to compare the efficacy of individual preparations due to differences across studies in all of the listed parameters.

Neurotoxins such as MPTP, paraquat, rotenone, and 6-OHDA were used as PD inducers and administered intraperitoneally, subcutaneously, or intranigrally. Neurotoxins are widely used to produce PD in animal models, triggering oxidative stress, neuronal loss, and neuroinflammation. The main signs of PD induced by these toxins are motor deficits caused by the almost complete loss of dopaminergic cells in the SN. Almost all studies conducted behavioral tests and noted that bacteria significantly improved motor deficits: motor performance and balance, movement coordination, and strength [96,97,100,101,102,103,104,105,108]. The positive effect of bacterial preparations was also noted in behavioral tests for cognitive deficits, various aspects of learning, memory, anxiety-like behaviors, and exploratory activity [103,104,107]. Almost all animal model studies of PD indicate that various bacterial preparations can positively affect dopaminergic neuron preservation and prevent neuronal damage in the SN [95,97,98,99,100,101,102,103,104,105,106,107]. An increase in brain dopamine [95,96,97,102,107] and acetylcholinesterase [96,97] levels has also been noted—two major neuromodulators regulating basal ganglia activity.

The root cause of altered dopaminergic neuron activity in PD is the agglomeration of misfolded protein aggregates known as α-synuclein in the brain and the gut. Bacterial preparations are capable of reducing the expression of the α-synuclein gene in the intestine [100], α-synuclein and tau proteins in the striatum [97] and SN [101], and the level of α-synuclein phosphorylation in the SN [108].

Neuroinflammation plays a critical role in PD progression and is mediated and facilitated by various pro-inflammatory cytokines. A growing body of evidence suggests that gut inflammation is the initiator of neuroinflammation. Neurotoxins used to model PD induce not only neuroinflammation but also gut and systemic inflammation. Bacterial preparations reduce inflammatory processes: the expression of pro-inflammatory cytokines decreases while anti-inflammatory cytokines increase in the brain [95,97,98,101,103,104,105,107]. The expression of the C-reactive protein is also reduced [104]. Glial activation decreases [98,101,105,107], and inhibition of the microglial NLRP3 inflammasome is observed [98]. Bacterial preparations restore levels of anti-inflammatory mediators GDNF and BDNF [95,107]. A reduction in inflammation due to bacterial preparations is also evidenced by changes in inflammatory markers in the brain: decreased levels of MDA [96,97,104], nitric oxide synthase [104], ROS [102], and increased antioxidant capacity via SOD, catalase, and GSH expression [96,97,104]. The anti-inflammatory effect of bacterial preparations is observed at both systemic and intestinal levels. They reduce the expression of pro-inflammatory cytokines and increase the expression of anti-inflammatory cytokines in the intestine and colon [95,98,107], the serum [95,103], and the liver [95], and inhibit the intestinal NLRP3 inflammasome [95]. Bacterial interventions improve BBB integrity [101,105,107].

The gut–brain axis plays a crucial role in the development of neurological and neurodegenerative disorders, including PD [1]. Gut-related symptoms are commonly observed in PD. Alterations in the composition of the gut microbiota and intestinal barrier permeability may contribute to the development of PD, at least in some subsets of the disease [67]. Neurotoxins used in animal models of PD, along with other physiological changes, significantly alter the composition and properties of the gut microbiota. The impact on microbiota was investigated in only five of the studies listed in Table 1. The results vary considerably. An improvement in microbiota richness and diversity was observed [101,107]. Both neurotoxin exposure alone and combined with probiotic administration led to changes in microbiota composition compared to control, but these changes did not compensate for each other. Changes induced by probiotics compared to neurotoxins at the phylum level differed between studies: a reduction in Firmicutes [101], an increased Firmicutes/Bacteroidetes ratio [102], and a reduction in Bacteroidetes [105]. At the family level, the following changes were noted: reductions in *Prevotellaceae* and *Desulfovibrio* [101]; *Alloprevotella*, *Prevotellaceae* Ga6a1 group (associated with anxiety-like behavior in rats), and *Lachnospiraceae* [103]; *Muribaculaceae*, *Bacteroides*, and *Prevotellaceae* [105]; and increases in *Oscillibacter*, *Lactobacillus*, *Butyricicoccus* (a butyrate producer) [102], *Akkermansia*, *Roseburia*, and *Dubosiella* [105]. While specific microbiota alterations varied, the overall effect was a reduction in pathogenic and pro-inflammatory taxa and the restoration of potentially beneficial bacteria.

The positive effects of bacterial preparations were also evident in the correlation between the relative abundance of specific fecal bacteria and certain traits: Bifidobacterium in feces showed a significant positive correlation with fecal levels of acetic and propionic acids; the relative abundance of the *Eubacterium ruminantium* group was significantly positively correlated with BDNF and GDNF expression in the striatum and TH-positive cells in the SN; and there was a negative correlation between fecal *Eubacterium ruminantium* group abundance and IL-6 and TNF-α expression in the striatum [107]. Analyzing bacterial metabolites may be an even more informative indicator. Bacterial intervention significantly increased the levels of beneficial bacterial metabolites. In feces of animals from the bacterial intervention group, compared to the PD group, levels of indole-3-acrylic acid, docosatrienoic acid, and indole-3-butyric acid increased, while levels of N-acetylhistamine, indole-3-acetic acid, and speridine decreased. A significant increase in SCFAs was also observed [107]. The increase in SCFAs in feces following bacterial intervention was confirmed in other studies as well [104,105].

Bacterial intervention also improves gastrointestinal motility [101,102,103,104,105], intestinal barrier integrity [102,104,107], and small intestine histology [103].

Bacterial strains such as *B. animalis* subsp. *lactis* NJ241 and *L. plantarum* SG5 enhance GLP-1 expression in the colon and increase serum GLP-1 levels, as well as GLP-1 receptor (GLP-1R) and PGC-1α expression in the SN of mice. GLP-1 (glucagon-like peptide-1) can promote insulin secretion, regulate gastrointestinal motility, and exert protective effects on the nervous system. The neuroprotective effects of bacterial preparations were attenuated when a GLP-1R antagonist was applied. These data indicate that the action of probiotics is at least partially mediated through the GLP-1/PGC-1α signaling pathway [101,105]. Additionally, genetically engineered bacterial strains delivering GLP-1 have also demonstrated neuroprotective effects in PD animal models [110,111].

Bacterial preparations are administered orally and primarily act on the gut microbiota, altering its composition, reducing the pathogenic component, and changing the spectrum of produced metabolites. The microbiota bacteria synthesize or facilitate the synthesis of metabolites in the host organism, such as neurotransmitters (dopamine, norepinephrine, serotonin, GABA, glutamate, and histamine), exopopolysaccharides, vitamins, antimicrobial peptides, secondary bile acids, and hydrogen sulfide [67]. Bacteria also produce SCFAs through microbial fermentation of dietary fiber in the colon, which are crucial for gastrointestinal and immune health [70]. In the studies reviewed, only some authors linked the activity of bacterial preparations to the production of specific products, specifically suggesting the synthesis of vitamins (riboflavin and folic acid) [103] and exopolysaccharides [102].

In a study of the *L. fermentum* U-21 strain, which exhibits anti-Parkinsonian activity [108], a 76 kDa protein was found in the cell-free supernatant and extracellular vesicles (EVs) of the strain, which was absent in the supernatant of the *L. fermentum* 279 strain that lacks such properties. This protein was identified as ClpL, the ATP-binding subunit of the Clp ATP-dependent protease. ClpL belongs to the HSP100 heat shock protein family and interacts with the DnaKJ/GrpE chaperone complex involved in the disaggregation of denatured proteins. In vivo in *E. coli* cells and in vitro, the ClpL protein demonstrated the ability to refold luciferase proteins. The cell-free culture fluid of the strain also had disaggregating activity [114]. Disaggregases are indeed being considered as potential drug candidates for the treatment of neurodegenerative diseases [115]. It is possible that the antioxidant and anti-Parkinsonian effects of the *L. fermentum* U-21 strain are partly due to the ClpL protein and its ability to refold protein aggregates.

The possibility of intranasal use of bacterial preparations based on commensal bacteria has also been demonstrated. Compared to the gut microbiota, bacteria of the nasal microbiota—especially those in the olfactory mucosa—are geographically closer to the brain and potentially have a tighter connection with the CNS via metabolite exchange. This offers additional advantages for the treatment of neurodegenerative diseases [116,117]. Recombinant commensal bacteria, particularly lactobacilli, can be used as intranasal drug delivery vehicles, highlighting their potential for brain-targeted therapies [118].

The nematode *C. elegans* is also used as a model to study the properties of functionally active bacteria [119]. Models of PD have been developed in *C. elegans*. In the study by Goya et al. [120], it was shown that diverse *B. subtilis* strains inhibit and clear human α-synuclein aggregation in transgenic *C. elegans* worms. The bacterium acts through metabolites and biofilm formation to activate protective host pathways, including DAF-16/FOXO and sphingolipid metabolism. In a more detailed study by Francisco and Grau [99], it was shown that *B. subtilis* strains used as gut commensals in *C. elegans* prevented the aggregation of GFP-tagged human α-synuclein in worms. To exert this effect, the strains needed to maintain biofilm formation proficiency and the QS peptide CSF. Biofilm-forming *B. subtilis* also protects against age-related and 6-OHDA-induced dopaminergic neurodegeneration, improves dopamine-dependent behaviors, and extends the lifespan of worms. The protection of *C. elegans* from 6-OHDA-induced dopaminergic neuron damage occurred not via insulin/IGF-1 signaling, as in the case of α-synuclein aggregation inhibition in the previous study, but through activation of PMK-1/SKN-1 signaling, which are major regulators of genes involved in detoxification and antioxidant defense. In the studies mentioned, the beneficial properties exhibited by *Bacillus* strains were not strain-specific but were shared by several strains capable of forming biofilms. It is likely that the ability to produce nitric oxide is also important for the probiotic effects of *Bacillus*. Previously, it was shown that the increased lifespan and stress resistance of *C. elegans* caused by *B. subtilis* were due to nitric oxide synthesized by the bacteria via NO synthase [121]. Lactobacilli are poorly suited to serve as gut commensals in *C. elegans*, but some strains have been found to increase the nematode’s lifespan after exposure to a neurotoxin [122].

All the reviewed studies clearly demonstrate that certain bacterial strains are capable of alleviating or even completely eliminating PD symptoms induced by neurotoxins, acting similarly to pharmaceutical drugs. The application of probiotic bacterial strains in PD therapy represents a highly promising direction. However, several important limitations must be considered. Probiotics are live microorganisms and, in rare cases, may trigger pathological inflammatory processes. This restricts their use in critically ill or immunocompromised patients, as well as in individuals with vascular catheters. Since most PD patients are elderly and often present with multiple comorbidities, these safety concerns are particularly relevant. Certain strains of *Lactobacillus* and *Bifidobacterium* produce both L- and D-lactate. The latter can accumulate in the intestine and cause D-lactic acidosis. In rare instances, probiotic intake has been linked to symptoms of D-lactic acidosis, such as brain fog or somnolence. Discontinuation of probiotics, in combination with antibiotic therapy, typically resolves acidosis and alleviates neurological symptoms [123,124]. Careful strain-specific characterization of candidate probiotic preparations is therefore essential. In addition, the development of postbiotics and paraprobiotics offers an important complementary strategy. Postbiotics (also called metabiotics) are metabolic products secreted by probiotics and their complex mixtures, while paraprobiotics are inactivated microbial cells of probiotics or their cellular components [125,126]. There are some data on the use of paraprobiotics in PD models. Heat-killed *L. murinus* improved 6-OHDA-induced motor impairments and prevented the loss of dopaminergic neurons in the SN, while live *L. murinus* showed no protective effect. The heat-killed bacteria also reduced NLRP3 inflammasome activation in microglia and the secretion of proinflammatory factors, thereby inhibiting neuroinflammation [127]. Certain bacterial cell components, such as SCFAs, spermine, spermidine, indole, and exopolysaccharides have neuroprotective effects in animal models [128]. EVs are of particular interest as postbiotics.

EVs are nanosized particles released from prokaryotic and eukaryotic cells, enclosed in a lipid bilayer. The cargo of bacterial EVs includes proteins, neurotransmitters, lipids, polysaccharides, phages, DNA, RNA, and microRNAs. Bacterial EVs perform a wide range of functions: exporting misfolded proteins and peptidoglycan fragments from cells; binding and transporting cytosolic metabolites, phages, antibiotics, and bioactive peptides; and transferring DNA. EVs mediate interactions between microbiome bacteria and host cells [129]. Bacterial EVs are absorbed by intestinal epithelial cells and nerve endings, enter the bloodstream, and cross the blood–brain barrier. EVs of Gram-negative pathogenic bacteria can shuttle bacterial toxins and virulence factors throughout the body, triggering inflammatory responses. There is evidence that these EVs may contribute to PD by initiating local, systemic, and neuroinflammation [130]. EVs from probiotic bacteria possess biological activity and are being considered as potential postbiotics [131,132]. Bacterial EVs can serve as vectors for the targeted delivery of specific metabolites and proteins to target cells. There is evidence that EVs from probiotic bacteria suppress neuroinflammation, reduce neuronal damage, and influence serotonin signaling [133]. In a model of Aβ phagocytosis in BV2 microglia, EVs from *B. coagulans* lilac-01 and *E. coli* DH5α significantly increased Aβ uptake in a dose-dependent manner [134]. EVs from *L. rhamnosus* ATCC 7469 reduced lipopolysaccharide-induced neuroinflammation in mice [135]. Lactobacillus-derived EVs have attracted significant attention due to their diverse physiological functions [136]. Biologically active proteins and metabolites have been identified in EVs from the strain *L. fermentum* U-21 [108], which may account for its biological activity [137,138]. In a study by Sheikh et al., 2025 [100], it was shown for the first time that EVs from the probiotic *L. acidophilus* PTCC 1643 reduce movement disorders and increase serotonin (5-HT) and TH levels in the SN in a 6-OHDA rat model of PD.

EVs are considered promising therapeutic agents because, unlike live bacteria, they carry a complex of bioactive proteins and metabolites and can be used as vectors for drug delivery.

### 4.2. Probiotics in Parkinson’s Disease: Human Studies

Animal models of PD have made it possible to identify bacterial strains and compositions with neuroprotective and anti-inflammatory effects, protecting animals from neurotoxins. These studies represent only the first stage in the search for probiotics aimed at alleviating PD symptoms. Due to the characteristics of PD (its long-term progression and unclear origin), there are no animal models that fully replicate the human disease.

Nevertheless, the success in identifying antiparkinsonian effects of bacterial preparations has led to their use in clinical trials. Results from these studies are summarized in Table 2.

The reviewed studies used various bacterial species: *S. thermophilus*, *Enterococcus faecium*, *B. coagulans*, and various *Lactobacillus* strains (*L. rhamnosus*, *L. acidophilus*, *L. plantarum*, *L. paracasei*, *L. casei*, *L. delbrueckii*, *L. reuteri*, and *L. fermentum*), as well as Bifidobacteria (*B. breve*, *B. animalis*, *B. infantis*, *B. bifidum*, and *B. longum*). It is not merely the species but the specific strain of bacteria that determines their effects on human health. In some studies, unfortunately, the strains were not specified. However, most studies did report the strain identity. Many of the strains used had already proven effective in previous studies unrelated to PD, such as *L. paracasei* strain Shirota [139,148], *B. animalis* subsp. *lactis* BS01, *B. longum* 03, *B. adolescentis* BA02 [152], *L. plantarum* PS128 [145,155], and *B. animalis* subsp. *lactis* Probio-M8 [147]. Commercial probiotic formulations were also used, such as Hexbio^®^ (B-Crobes Laboratory, Malaysia) [143], Symprove (Symprove Ltd.) [144], Enterolactis Duo L. paracasei DG (DSM 34154) [151], Comflor^®^ (Fara Daroo Fanavar Mehr Co) [150], BioZen D [153], and Livia^®^ (Pharma Zad, Egypt) [154]. Prebiotics such as inulin, fructooligosaccharides, and vitamins D and B-group were also used. It is worth noting that the *L. paracasei* (Shirota) strain was successfully tested in two studies, and *L. plantarum* PS128 showed positive results both in clinical trials and animal experiments. The typical duration of probiotic administration was 3 months, with high daily doses (10^9^–10^10^ CFU).

The conducted studies were predominantly randomized, double-blind, placebo-controlled clinical trials (RCTs). The number of participants ranged from 25 to 128, with average participant age between 56 and 76 years. All studies included both men and women. The probiotic treatments lasted between 4 and 12 weeks, most commonly 12 weeks. Probiotics were administered in the form of fermented milk, capsules, or powder sachets. In all studies, probiotics were used alongside standard medications (levodopa, dopamine agonists, and MAO-B inhibitors).

The functional bacteria were generally well tolerated by patients, with rare and mild adverse effects. For instance, Ibrahim et al. [43] reported two cases of abdominal symptoms and two of dizziness; Tan et al. [146] mentioned one case of lethargy after probiotic treatment.

All studies reported improvements in PD patients’ health parameters, though the nature of these improvements varied. Regarding patient condition and behavior, general improvement in quality of life [145,149,153] prolonged ON periods, and reduced OFF periods [145] were noted.

Constipation is a predominant NMS of PD, often appearing years before motor symptoms. Its pathophysiology involves multiple factors: PD-related GI dysfunction, side effects of antiparkinsonian medications, sedentary lifestyle, reduced intestinal motility, and impaired anal sphincter function. Probiotics have proven effective in treating constipation in non-PD adult patients [156]. In PD-related probiotic studies, the following improvements were observed: improved stool frequency [140,143,146,147,148,150,151], improved whole gut transit time [145], and improved stool consistency [139,147,148,150,151]. These findings are supported by reduced laxative use [140]. However, some studies did not show statistically significant improvements in constipation symptoms [141,152].

An analysis of data from a single patient with recently diagnosed PD, who began a diet enriched with plant-based foods and dietary fiber, along with probiotic supplementation six months after diagnosis, showed a rapid disappearance of constipation symptoms. It is possible that the use of probiotics is most effective in the early stages of the disease [157].

Probiotic intake also decreases gastrointestinal problems such as bloating and abdominal pain [139,141,151,152,153]. The effects of probiotics on the gastrointestinal tract in PD patients may be multifaceted, including general improvements in well-being and possibly influencing the formation of synuclein aggregates. Furthermore, by promoting intestinal transit, probiotics may enhance the absorption of PD therapy, thereby increasing its clinical effectiveness.

Many studies have also noted improvements in other NMS. These include enhanced cognitive function [147,151,152], reductions in alexithymia and dysautonomia [151], improved sleep quality [147], and improvements in mental health parameters such as depression, anxiety, and panic disorder [147,148,149,151,153]. The effects of different probiotic strains on depression and anxiety symptoms have been studied previously [158,159].

Some studies report improvements in motor function, UPDRS-III scores, and akinesia [145,147,152]. However, other studies did not find improvements in motor symptoms with probiotic use [143,150,151].

Biochemical studies have also been conducted. Investigations among PD patients demonstrated a reduced endogenous antioxidant system [35]. In the studies reviewed, probiotic intake increased total antioxidant capacity (TAC) [149,153], reduced oxidative stress index [149], and improved markers of oxidative stress in serum—lowering levels of malondialdehyde [149,153,154] and myeloperoxidase [145]. In the study by Borzabadi et al. [142], however, no change in GSH levels was observed with probiotic intake.

Neuroinflammation is one of the key factors in PD pathogenesis, along with alpha-synuclein accumulation, synaptic dysfunction, and dopaminergic neuronal loss. Aggregated alpha-synuclein binds to Toll-like receptors and activates microglia, initiating the production and secretion of pro-inflammatory cytokines [160]. Regulating inflammatory markers may reduce central inflammation and help restore nervous system functions. Probiotic intake may reduce gene expression levels of inflammatory markers [161]. Studies have reported downregulation of pro-inflammatory cytokines (IL-1β, IL-8, IFN-γ, TNF-α) in peripheral blood mononuclear cells [142] and serum [152,153,154], as well as upregulation of the anti-inflammatory cytokine IL-10 in serum [153].

Probiotics may also affect glycemic status and insulin sensitivity. In animal studies, insulin resistance increased after probiotic use [162]. In clinical trials, probiotics reduced blood glucose levels [163]. Among the studies reviewed, only one investigated this aspect. These reported upregulation of peroxisome proliferator-activated receptor gamma in peripheral blood mononuclear cells [142]. This receptor, which acts as a transcription factor, is involved in fatty acid storage and glucose metabolism [164].

Other serum markers also changed—there was an increase in acetic acid and dopamine and a decrease in glutamine and tryptophan [147]. Yang et al. noted an increase in serum tyrosine and a decrease in fecal tyrosine [148]. Probiotics may improve impaired digestion and absorption of L-tyrosine in the upper digestive tract, increasing its level in the serum. A statistically significant increase in the butyrate/acetate ratio in fecal samples was also observed after synbiotic intake [151].

The primary action of probiotics is on the gut microbiota, and understanding how it changes in response to probiotics is essential. Unfortunately, only four of the reviewed studies examined microbiota composition. These studies reported that probiotic supplementation did not cause drastic changes in gut microbiota diversity or structure [147,148], though it did alter the microbiota composition. These changes likely occur rapidly: in an in vitro gut model using PD patient microbiota, changes in microbial composition and an increase in SCFAs and lactate production were observed within 48 h of probiotic addition [144]. The specific changes varied across studies: suppression of *L. fermentum* and potentially pathogenic taxa (*Klebsiella*) and increases in beneficial taxa (*Ruminococcaceae*, *Lachnospira*, *Butyricimonas*) [147]; increased *Lacticaseibacillus* [148]; and increases in *Oscillospiraceae*, particularly *F. prausnitzii*, a major butyrate producer, along with an increase in the butyrate/acetate ratio in fecal samples [151]. In the same in vitro model, probiotic dosing improved colonic media tight junction integrity. The probiotic itself did not directly affect tight junctions—rather, the effect was mediated by the altered microbiota and increased SCFA levels [144].

A number of studies have conducted systematic reviews and meta-analyses of existing research on the effects of probiotics in patients with PD. These analyses include various publications, and the conclusions drawn by authors are not always consistent. Overall, such studies confirm the effectiveness of probiotics in improving PD-related symptoms such as constipation [156,165,166,167,168], UPDRS-III motor scores [166,169], and depression [169]. These studies also highlight that the efficacy of probiotics appears to be greater when administered in capsule form compared to fermented milk [156,169].

Although early clinical trials investigating bacterial preparations in PD patients have yielded encouraging results, further research is essential. It should be noted that the number of available clinical studies remains limited; most involve small patient cohorts and report heterogeneous outcomes. Large-scale trials with well-defined, homogeneous patient groups are urgently needed. Early intervention and long-term administration of bacterial products may prove especially beneficial in slowing disease progression and alleviating symptoms. In particular, detailed investigation of bacterial formulations and the development of live biotherapeutic products (LBPs) based on them is necessary. This includes identifying suitable PD models, exploring novel probiotics and postbiotics—especially bacterial vesicles—and searching for early PD biomarkers [170,171,172].

Table 3 provides examples of bacterial preparations aimed at treating neurodegenerative diseases, including PD.

An important but underexplored microbial intervention for PD is fecal microbiota transplantation (FMT). FMT involves the transfer of fecal material from a healthy donor into the gastrointestinal tract of a patient via colonoscopy, gastroscopy through a nasojejunal tube, or oral capsules containing the microbiota. Unlike probiotics, FMT delivers a complex microbial community rather than a single strain. FMT has proven highly effective in treating recurrent *Clostridium difficile* infections [177] and is occasionally used in other conditions linked to gut dysbiosis. In rodent models of PD, FMT was shown to correct dysbiosis, reduce inflammation in the gut and brain, and alleviate disease symptoms [178,179]. To date, four randomized controlled trials have been published examining the effects of FMT in PD patients [180,181,182,183]. The study methodologies and outcomes varied, but in general, mild-to-moderate adverse events were transient and gastrointestinal in nature. Modest but statistically significant improvements were observed in gut motility/constipation, motor and NMS, and overall PD severity scores [67,184]. However, such studies are extremely difficult to standardize. Outcomes depend heavily on the microbiota profiles of both patients and donors, the method and formulation of FMT administration, and the quantity of transplanted material. Additionally, the potential risk of serious infections associated with FMT should not be underestimated.

## 5. Neurodegenerative Diseases—Parkinson’s Disease—The Potential of Machine Learning for Diagnosis and Treatment

In recent years, artificial intelligence (AI) technologies—particularly neural networks and machine learning—have entered nearly all areas of science, including biomedicine. Disorders of the nervous system, such as PD, have increasingly drawn the attention of AI researchers. Several publications have applied machine learning approaches to classify different forms of PD, and attempts have been made to refine biomarkers specific to PD [185]. Other studies have proposed machine learning–based methods to support PD diagnosis [186].

**Figure 3:**
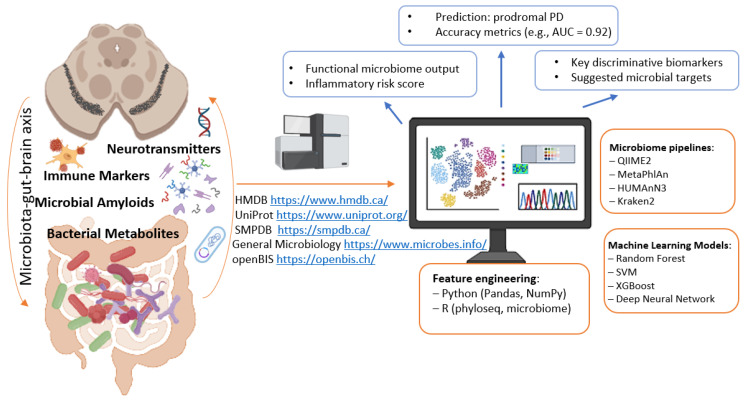
Digital parameters of the microbiome—indicators for intervention. Parts of this figure were created in BioRender (2025) https://BioRender.com/ocetej3 (accessed on 1 July 2025).

A vast amount of information generated from gut microbiome studies and digitized in databases cannot be effectively analyzed without the use of AI technologies (Figure 3). The integration of omics approaches, including metagenomic sequencing, with modern machine learning methods for studying the microbiota of both healthy individuals and patients with neurological disorders provides unique insights into the functional characteristics of microorganisms. In this context, machine learning can serve as an in silico diagnostic tool, as well as a means of identifying key microbiota biomarkers and therapeutic targets. Machine learning methods are increasingly applied to microbiota data to predict the risk of developing neurodegenerative diseases [187]. Distinct microbiota profiles have already been identified in patients with PD [188,189]. Several studies have reported the application of machine learning to predictive modeling of microbiome biomarkers (at the bacterial genus level) based on comparative analyses of cohorts of PD patients and healthy individuals [64,190].

**Figure 4:**
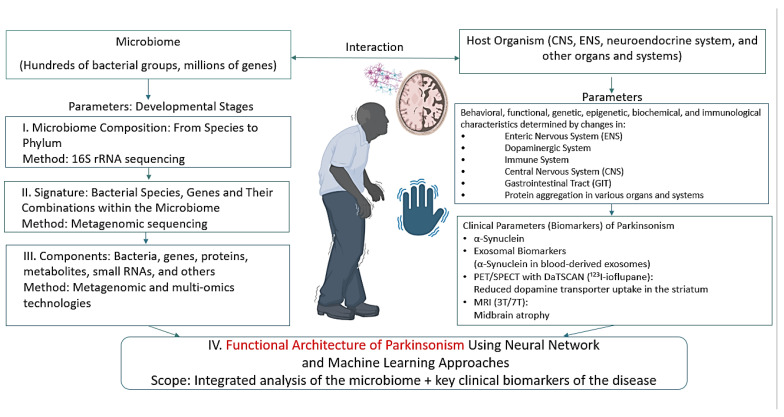
Parameters characterizing the microbiome–host organism system in PD. Parts of this figure were created in BioRender (2025) https://BioRender.com/vs5aep9 (accessed on 1 July 2025).

Research combining neural networks and machine learning in the context of PD appears promising in two main directions: first, in the development and analysis of neural networks that integrate and model the functional architecture of PD; a schematic overview of relevant biomarkers and algorithms for constructing such networks is presented in Figure 4, with methodological approaches described in [191]; second, machine learning can be applied to mine gut microbiome databases for pharmabiotics and their metabolites with targeted antiparkinsonian effects—similar to how such approaches have already been employed to identify microbiome-derived pharmabiotics with antidepressant properties [158,192,193].

## 6. Conclusions

PD is a complex, multifactorial disease, both in terms of its clinical manifestations and the range of organs and systems it affects during development and progression. Currently, no drugs are available that can cure the disease. Medications used in clinical practice generally target only one of the systems affected. Drug development strategies evolve with advances in chemistry, biochemistry, genetics, molecular biology, systems biology, bioinformatics, mathematical modeling, AI (including machine learning), and related fields.

Over the past decades, target-specific drugs have been developed—sometimes successfully—through chemical synthesis or by isolation from animal and plant sources, soil bacteria, and other origins. PD, like other neurodegenerative disorders, is characterized by dysfunction across multiple biological targets within the networks that maintain overall homeostasis. Based on this understanding, effective drugs for PD should ideally consist of combinations of pharmacologically active substances in precise compositions and ratios.

The biological targets of these drugs are already partially known and are expected to be further refined. Insights into the mechanisms underlying PD and the affected targets may also be gained from studies on post-COVID PD [194,195]. A promising source of such drugs is the microbiota of healthy individuals—a stably balanced system that supports homeostasis, even under stress.

Which microbiota-based bacteria are suitable for developing drugs targeting PD mechanisms? These should be functional bacteria-possessing genes and their products in combinations that match the biological targets they are intended to influence. This is supported, in part, by the demonstrated ability of certain probiotics to exhibit properties relevant to PD therapy. The next step involves selecting LBPs based on these strains, which are considered medicinal products under modern standards [196]. Postbiotics and vesicle-based preparations may also be promising therapeutic options.

LBPs and their derived vesicles can serve as carriers for proteins, low-molecular-weight metabolites, neurotransmitters, immunomodulators, anti-inflammatory agents, and small RNAs capable of modulating gene and receptor activity, including in neurons and immune cells. These interventions may, for example, promote the production of antibodies against misfolded proteins such as α-synuclein.

The human microbiome represents a vast resource for developing nature-inspired medicinal products for PD therapy. Such drugs have the potential to eliminate pathogenic gut bacteria, restore intestinal barrier integrity, recover bacterial communities that produce neurotransmitters, neurohormones, and their precursors, halt α-synuclein formation in both the enteric and central nervous systems, neutralize free radicals and reduce oxidative stress, and alleviate associated depressive symptoms and cognitive dysfunctions.

## Figures and Tables

**Table 1:** Investigations of bacterial interventions in Parkinson’s disease animal models (2024–2025).

No.	Microbial Intervention	Animal	BP Model Inductor	Duration of the Experiment; Dose of Bacteria per Animal per Day	Conducted Research	Result	Target in the Animal’s Body	Active Components of Bacteria	References
1	VSL#3	C57BL/6 mice; 6 mice/group	MPTP I.P. 30 mg/kg 5 days	6 weeks; 4 × 10^9^ CFU	1. In striatum	1. DA↑,DOPAK↑, NVA↑, NE no changes	Inhibition of the intestinal NLRP3 inflammasome		[95]
					2. In SN	2. TH+ neurons↑			
					3. In striatum	3. GDNF↑, BDNF↑, TNF-α↓, IL-β↓			
					4. In serum	4. TNF-α↓, IL-1β↓, IL-6↓, IL-17↓, IFN-γ↓, GM-CFS↓			
					5. In liver	5. TNF-α↓, IL-1β↓			
					6. In intestine	6. TNF-α↓, IL-1β↓, IL-6↓, IL-17↓, caspase-1↓, NLRP3↓			
2	*L. rhamnosus* (Unique Biotech Ltd., Telangana, India) + curcumin 500 mg/kg	Sprague Dawley rats; 6 rats/group	Rotenone S.C. 2.5 mg/kg 3 weeks	3 weeks; 0.1 × 10^9^– 1 × 10^9^ CFU	1. Behavioral tests	1. Hanging wire↑, akinesia↓, catalepsy↓			[96]
					2. Brain homogenate	2. DA↑, AchE↓, SOD↑, catalase↑, GSH↑, MDA↓			
					3. Histopathology of brain sections	3. Improvement in the histology of the brain.			
3	*L. fermentum*, *L. delbrueckii*, +vinpocetine 20 mg/kg	Sprague Dawley rats; 10 rats/group	Rotenone I.P. 2.5 mg/kg	8.5 weeks	1. Behavioral tests	1. Motor symptoms improved in movement coordination and strength.			[97]
					2. In striatum	2. DA↑, GSH↑, nitrite↓, MDA↓, IL-1↓, TNF-α↓, α-synuclein↓ tau↓			
					3. In SN	3. TH+↑			
4	*A. municiphila* Akk11	C57BL/6 mice; 8 mice/group	MPTP 30 mg/kg +probenecid 250 mg/kg I.P. 6 days.	4 weeks, 10^9^ CFU	1. Behavioral tests	1. open field↑, pole↓, rotarod↑ tests	Inhibition of microglial TLR4/NF-κB/NLRP3 inflammasome activation		[98]
					2. SN	2. TH+ ↑ TH protein expression↑			
					3.Expression of cytolines in SN	3. IL-1β↓, TNF-α↓, IL-6↓, TGF-β↑, IL-10↑, Arg-1↑			
					4. Microglia and the NLRP3 inflammasome	4. Activation of microglia ↓			
					5. Colon	5. Colonic integrity↑, IL-1β↓, TNF-α↓, IL-6↓.			
5	*B. subtilis* NCIB3610	*Caenorhabditis elegans*	6-OHDA 75 mM		1. Dopaminergic neuron .	1. Dopaminergic neuron injury↓	PMK-1 (p38 MAPK)/SKN-1 (Nrf2) signaling	Bacterial biofilm (hydrophobic BskA protein) and QS peptide CSF	[99]
					2. Human alpha-synuclein in transgenic worms	2. Aggregation of alpha-synuclein↓			
6	*L. acidophilus* PTCC 1643, membrane vesicles	Wistar rats; 5 rats/group	6-OHDA bilateral injection in SN; The rats rested for one month after surgery and entered the study after PD was confirmed	4 weeks, 3 d/wk; equivalent 1 × 10^7^ CFU	1. Behavioral test.	1. Beam-walking test ↑			[100]
					2. SN	2. Protein and genes (receptor) of 5-HT ↑, protein and gene (receptor) of GABA ↓, TH ↑			
					3. The intestine	3. The expression of TLR-4 and α-synuclein gene ↓			
7	*L. plantarum* SG5	C57BL/6 mice; 8 mice/group	MPTP I.P. 30 mg/kg 5 days	5 weeks; 1 × 10^9^ CFU	1. Behavioral tests	1. Rotarod↑, hanging test↑	The GLP-1/PGC-1α signaling pathway		[101]
					2. SN	2. Dopaminergic neuron count↑, TH↑ α-syn↓, activation of glial cells↓ NF-κB↓ IL-1β↓, ZO-1↑, GLP-1R↑ PGC-1α+↑.			
					3. BBB	3. BBB integrity↑			
					4. Gut and colon	4. Gut microbiota richness↑ diversity↑, intestinal transit↑, intestinal barrier integrity (ZO-1↑, occludin↑)↑, NF-κB↓, IL-6↓, IL-1β↓, GLP-1↑.			
					5. Serum	5. GLP-1↑			
8	*L. rhamnosus* E9	C57BL/6 mice; 15 mice/group	MPTP I.P. 30 mg/kg 5 days	2 weeks; 10^8^ CFU	1. Behavioral tests.	1. Open field↑, catalepsy↓, wire hanging test↑		EPS	[102]
					2. Striatum	2. TH gene and protein↑, DA↑, *DR1*↓, *DAT*↑, ROS↓			
					3. Gut	3. Occludin gene and protein↑, remodulation of the cecal microbiota at the phylum and genus level, Firmicutes/Bacteroidota↑			
9	*L. plantarum* CRL2130, *S. thermophilus* CRL808, *S. thermophilus* CRL807	C57BL/6 aged (1-year-old) mice; 7 mice/group	MPTP I.P. 20 mg/kg +Probenecid I.C. 250 mg/kg	6 weeks; 1.8 ± 2 × 10^7^ CFU of every strain	1. Behavioral tests.	1. Pole test↓, transversal beam test↓,, nasal bridge adhesive removal test↓, foot sliding test↓		Vitamins: riboflavin, folic acid	[103]
					2. SN	2. TH + cells↑			
					3. Brain	3. IL-6↓, TNF-α↓, IL-10↑			
					4. Serum	4. IL-6↓, TNF-α↓, IL-10↑			
					5. Gut and small intestine	5. Villi length/crypts depth↑, dysbiosis↓ (Lactobacillceae↑ Prevotellacea↑Alistipes↑)			
10	*B. breve* Bif11	Sprague Dawley rats; 3–9 rats/group	MPTP 100 μg in SN	3 weeks; 1–2 × 10^10^ CFU	1. Behavioral tests	1. Y-maze spontaneous alteration↑, novel object recognition↑, rotarod↑, passive avoidance↑.			[104]
					2. Midbrain	2. TH ↑, MDA↓, GSH↑, nitrite↓ iNOS↓, IL-6↓, IL-1β↓, NF-κB↓, CRP↓			
					3. Feces	3. SCFA↑			
					4. Intestine	4. Intestinal epithelial permeability↑.			
11	*B. animalis* subsp. *lactis* NJ241	C57BL/6 mice	MPTP I.P. 30 mg/kg	4 weeks; 1 × 10^9^ CFU	1. Behavioral Tests	1. Open field↑, wire hanging ability↑	GLP-1R/PGC-1α signaling		[105]
					2. SN	2. TH+ cells↑, TH protein↑, glial activation↓, IL-1β, NF-κB, GLP-1R↑, PGC-1α↑			
					3. BBB	3. ZO-1↑ occludin ↑ in SN			
					4. Gut and colon	4. Gastrointestinal motility↑, gut dysbiosis↓, GLP↑			
					5. Feces	5. SCFA↑			
					6. Serum	6. GLP↑			
12	*B. animalis* subsp. *lactis* MH-022	Rat	6-OHDA		1. Behavioral Tests	1. Motor deficits↓	Mitochondria	SCFAs	[106]
					2. SN	2. Dopaminergic neurons↑, antioxidant capacity↑, inflammation↓			
					3. Gut	3. Normalization of the gut microbiota composition, SCFA↑.			
13	*B. longum* subsp. *longum* C7 CCFM1029	C57BL/6 J mice; 10 mice/group	MPTP I.P. 30 mg/kg	6 weeks; 5 × 10^8^ CFU	1. Behavioral Tests	1. Pole↓, beam walking↓, rotarod↑, open field↑	The modification of the gut microbiota and microbially produced metabolites.		[107]
					2. Striatum	2. LDOPA↑, DA↑, DOPAC↑, 5-HT↑, 5-HIAA↑, HVA↑, BDNF↑, GDNF↑, GFAP↓, Iba1↓, TNF-α↓, IL-1β↓, IL-6↓, ZO-1↑, occludin↑, claudin-1↑			
					3. SN	3. TH+↑			
					4. Gut and colon	4. TNF-α↓, IL-1β↓, IL-6↓, ZO-1↑, occludin↑, claudin-1↑, indole-3-acetic acid↓, spermidine↓, N-acetylhistamine↓, 3-acrylic acid↑, docosatrienoic acid↑, indole-3-butyric acid↑, SCFA↑, gut dysbiosis↓			
14	*L. fermentum* U-21	Wistar rats; 6/8 rats/group	Paraquat I.P. 8 mg/kg; LPS I.N. 4 mg/kg	2.5 weeks; 1 × 10^8^ CFU	1. Behavioral tests	1. Beam walking↓.			[108]
					2. SN	2. Phosphorylated α-synuclein↓, inflammatory glial response↓, complement component C3↓.			

**Abbreviations:** 5-HIAA—5-hydroxyindole acetic acid; 5-HT—hydroxytryptamine; 6-OHDA—6-hydroxydopamine; AchE—acetylcholinesterase; BBB—blood–brain barrier; BDNF—brain-derived neurotrophic factor; CRP—C-reactive protein; DA—dopamine; DR—dopamine receptor; DAT—dopamine transporter protein; DOPAC—3,4-dihydroxyphenylacetic acid; GABA—gamma-aminobutyric acid; GDNF—glial cell line-derived neurotrophic factor; GFAP—Glial fibrillary acidic protein; Glp-1—glucagon-like peptide; GSH—reduced glutathione; HVA—homovanillic acid; IN—intranigral; iNOS—nitric oxide synthase; IP—intraperitoneally; MDA—malondialdehyde; NE—norepinephrine; P.O.—orally; QS—quorum sensing; ROS—reactive oxygen species; S.C.—subcutaneously; SN—substantia nigra; SOD—superoxide dismutase; TH—tyrosine hydroxylase; TLRs—toll-like receptors; ZO-1—tight junction protein; ↑-increase; ↓-decrease.

**Table 2:** Randomized controlled trials of bacterial interventions for the treatment of Parkinson’s disease.

**N**	Type of Research	Composition and Form of Probiotic	Number of Patients; Average Age (Experiment/Placebo)	Duration of the Experiment	Conducted Research	Result	References
1.		*L. casei* Shirota 6.5 × 10^9^ CFU; fermented milk	40	5 weeks	Stools consistency, bloating, abdominal pain	Stools consistency↑, bloating↓, abdominal pain↓	[139]
2.		*S. salivarius* subsp *thermophilus*, *E. faecium*, *L. rhamnosus* GG, *L. acidophilus*, *L. plantarum*, *L. paracasei*, *L. delbrueckii* subsp *bulgaricus*, *B. breve*, *B. animalis* subsp *lactis*, 2.5 × 10^11^ CFU + prebotic fiber; fermented milk	80/40; 72/69 years	4 weeks	Rome III–confirmed constipation	Number of complete bowel movements per week (CBM)↑	[140]
3.		*L. acidophilus*, *B. infantis;* tablets	20 probiotic/ 20 trimebutine; 70/76 years	12 weeks	NMS-GI	Probiotics improve abdominal pain and bloating as much as trimebutine, but slightly less for constipation with incomplete evacuation.	[141]
4.	RCT	*L. acidophilus*, *B. bifidum*, *L. reuteri*, *L. fermentum*, 8 × 10^9^ CFU	25/25; 67/67 years	12 weeks	In peripheral blood mononuclear cells of PD patients: IL-1, IL-8, TNF-α, TGF-β, PPAR-γ, LDLR, VEGF; Plasma NO and GSH	IL-1↓, IL-8↓, TNF-α↓, TGF-β↑, PPAR-γ↑	[142]
5.	RCT	Hexbio^®^ *L. acidophilus* BCMC1 12130, *L. casei* BCMC1 12313, *L. lactis* BCMC1 12451, *L. lactis* BCMC1 02290, *B. infantis* BCMC1 02129, *B. longum* BCMC1 02120, 3 × 10^10^ CFU; +FOS; sachets (B-Crobes Laboratory, Perak, Malaysia)	22/26; 69/70 years	8 weeks	Garrigues Questionnaire (GQ) bowel opening frequency (BOF)	BOF↑	[143]
					gut transit time (GTT)	GTT↓	
					PDQ39-SI	no changes	
					UPDRS-II, UPDRS-III	no changes	
					NMSS	NMSS↓	
6.		Symprove *L. acidophilus* NCIMB 30175, *L. plantarum* NCIMB 30173, *.L. rhamnosus* NCIMB 30174, *E. faecium* NCIMB 30176 (Symprove Ltd., Surrey, UK)	3 patients; stool samples in an in vitro gut model	48 h	1. Colonic media from in an in vitro gut model in cell culture models— effect on epithelial tight-junction integrity, wound healing, production of inflammatory markers.	1. Tight junction integrity improved, wound healing was seen to occur faster, MCP-1↓ IL-8↓ IL-6↑ IL-10↑	[144]
					2. Bacterial composition and metabolic activity in the microbiotas of an in vitro gut model	2. Change in bacterial composition in the microbiotas; promotion of SCFA and lactate production	
7.	Single-arm, baseline-controlled trial	*L. plantarum* PS128 (PS128) 6 × 10^10^ CFU; capsules (Bened Biomedical Co., Ltd., Taipei, Taiwan)	25; 61 years	12 weeks	ON and OFF period duration	ON↑ OFF↓	[145]
					UPDRS	UPDRS total,↓ UPDRS-III↓	
					PDQ-39	PDQ-39↓	
					PGI-C	PGI-C (17 patients)↑	
					NMSS	no changes	
					PAC-SYM	no changes	
					BDI-II	no changes	
					Metabolic parameters	plasma myeloperoxidase↓, urine creatinine↓	
8.	RCT	Multistrain probiotic, 10 × 10^9^ CFU; capsules	34/38; 71/69 years	4 weeks	Number of spontaneous bowel movements (SBM)	SBM↑	[146]
					stool consistency	stool consistency↑	
					quality of life	quality of life↑	
					fecal calprotectin	no changes	
9.	RCT	Probio-M8 *B. animalis* subsp. lactis 3 × 10^10^ CFU; sachets	50/50; 69 years	12 weeks	PAC-QOL, UPDRS-III, MMSE, PDQ-39, HAMA, PDSS, HAMD-17, GI-related symptoms	PAC-QOL ↓, UPDRS-III ↓, MMSE↑, PDQ-39↓, HAMA↓, PDSS↑, HAMD-17↓, GI-related symptoms↓	[147]
					gut microbiome	Change in gut microbiome composition	
					7 kinds of SCFAs in serum	acetic acid ↑	
					12 types of neurotransmitters in serum	dopamin↑, glutamine↓, tryptophan↓	
10.	RCT	*L. paracasei* strain Shirota 10^10^ CFU; fermented milk.	65/63 67/70 years	12 weeks	Constipation	constipation-related symptoms↓	[148]
					NMSS	NMSS↓	
					HAMD-17	HAMD-17↓	
					HAMA	HAMA↓	
					PDQ-39	PDQ-39↓	
					fecal microbiota composition	Lacticaseibacillus↑	
					faecal metabolites	L-tyrosine↓	
					serum metabolites	L-tyrosine↑	
11.	RCT	*L. acidophilus* LAA-5, *L. rhamnosus* LAR-7, *L. plantarum LAP-10*, *B. longum* BIA-8, *S. thermophilus*, 5 × 10^9^ CFU +inulin; sachets.	40/40; 68/69 years	12 weeks	Serum biomarkers of oxidative stress	TAC↑ OSI↓ MDA↓ GSH no changes	[149]
					PDQ-39	PDQ-39↓	
					mental status	BDI-II↓ HADS no changes, FSS no changes	
12.	RCT	Comflor^®^ *L. plantarum*, *L. casei*, *L. acidophilus*, *Lactobacillus bulgaricus*, *B. infantis*, *B. longum*, *B. breve*, *S. thermophilus*, total of 4.5 × 10^11^ CFU; capsules (Fara Daroo Fanavar Mehr Co., Tehran, Iran).	14/13; 68/68 years	8 weeks	Defecation	Frequency of defecation↑	[150]
					Sense of complete evacuation	no changes	
					Bristol stool consistency	Bristol stool consistency↑	
					UPDRS	no changes	
13.	A single-arm, open label study	Enterolactis Duo *L. paracasei* DG (DSM 34154) 8 × 10^9^ CFU + inulin, vitamins B1, B2, B6; 4 times/day; sachets	30 65 years	12 weeks	MDS-UPDRS	MDS-UPDRS I-1, I-6, I-11↓	[151]
					SCOPA-AUT	SCOPA-AUT↓	
					TAS-20	TAS-20↓	
					HAM-D	HAM-D↓	
					DIFt	DIFt↓	
					PAS-A	PAS-A↓	
					PAC-SYM	PAC-SYM↓	
					STAI-Y	no changes	
					MoCA	no changes	
					RMET	no changes	
					BDI-II	no changes	
					MDS-UPDRS II, III, IV	no changes	
					fecal microbiota composition	Changes in the abundance of 8 taxa, (the genus *Faecalibacterium*↑)	
					fecal microbiota metabolites	butyrate/acetate↑	
14.	Preliminary in vivo data	*B. animalis* subsp. *lactis* BS01, *B. longum* 03, *B. adolescentis* BA02 ≥ 1 × 10^9^ CFU each + FOS	20/20; 68 years	12 weeks	UPDRS	UPDRS-III ↓	[152]
					NMSS	NMSS↓ (particularly in gastrointestinal symptoms)	
					plasma level of cytokines	IL-6↓, TNF-α no changes, IFN-γ no changes, TGF-β no changes	
15.	RCT	BioZen D *L. acidophilus*, *L. rhamnosus*, *L. reuteri*, *L. paracasei*, *B. longum*, *B. coagulans* 2 × 10^9^ CFU + vitamin D; capsules	23/23; 56/56	12 weeks	BAI	BAI↓	[153]
					GSRS	GSRS↓	
					UPDRS	UPDRS I, III, IV, total↓	
					serum level of cytokines and antioxidants	IFN-γ↓, IL-6↓, IL-1β↓, MDA↓, IL-10↑, TAC↑	
16.	RCT (open-label design)	Livia^®^ *L. acidophilus* 10^9^ CFU + inulin 3 g; twice a day; sachets (Pharma Zad, Cairo, Egypt)	33/33 45–65 years	12 weeks	MDS-UPDRS	MDS-URDS-I↓	[154]
					serum levels of cytokines and BDNF	TNF-α↓, MDA↓, BDNF↑	

**Abbreviations:** BAI—Beck anxiety inventory; BDI-II—Beck’s Depression Inventory-II; FSS—fatigue severity scale; FOS—fructo-oliogosaccharide; GSH—reduced glutathione; GSRS—Gastrointestinal symptom rating scale; HADS—Hospital Anxiety and Depression Score; HAMA—Hamilton Anxiety Scale; HAMD—Hamilton Depression Scale; hsCRP—C-reactive protein; LDLR—low-density lipoprotein receptor; MDA—Malondialdehyde; MoCA—Montreal Cognitive Assessment; MMSE—Mini-Mental State Examination; NMSS—Non-Motor Symptoms Scale; NMS-GI—Non-Motor gastrointestinal symptoms; OSI—oxidative stress index; PAC-SYM—patient assessment of constipation symptom; PAS—Parkinson Anxiety Scale; PDQ-39—39-item Parkinson’s Disease Questionnaire; PDSS—Panic Disorder Severity Scale; PGI-C—Patient Global Impression of Change; PPAR-γ—peroxisome proliferator-activated receptor gamma; RMET—Reading the Mind in the Eyes Test; SCOPA-AUT—Scales for Outcomes in Parkinson’s disease—Autonomic Dysfunction; STAI-Y—State–Trait Anxiety Inventory; TAC—total antioxidant capacity; TAS-20—Toronto Alexithymia Scale; UPDRS—Unified Parkinson’s Disease Rating Scale; VEGF—vascular endothelial growth factor. ↑- increase; ↓- decrease.

**Table 3:** Developers and manufacturers of LBPs aimed at the treatment of Parkinson’s disease and other neurodegenerative disorders.

	Purpose	Drug/Active Agent	Research Stage	Developer/Manufacturer
1	Parkinson’s disease	AB-202 (Microbiome-directed product)	Preclinical studies	Axial Biotherapeutics (USA) [173]
2	Parkinsonism and other neurodegenerative/neuroinflammatory conditions	PS128 (*L. plantarum* PS128)	Preclinical studies	Bened Biomedical (Taiwan) [174]
3	Neurodegeneration	MRx0029, (LPB) MRx0005 (LPB)	Preclinical studies	4D Pharma (United Kingdom) [175]
4	Neurodegenerative/neuroinflammatory diseases	ADS024 (*Bacillus velezensis* ADS024)	Preclinical studies	Adiso therapeutic (USA) [176]

## Data Availability

The original contributions presented in the study are included in the article; further inquiries can be directed to the corresponding author.

## Abbreviations

The following abbreviations are used in this manuscript:

6-OHDA	6-hydroxydopamine
BBB	Blood-Brain Barrier
CFU	Colony Forming Units
CNS	Central Nervous System
COMT	Catechol-O-methyltransferase
ENS	Enteric Nervous System
EVs	Extracellular Vesicles
FMT	Fecal Microbiota Transplantation
GABA	Gamma-Aminobutyric Acid
GSH	Reduced Glutathione
IL-6	Interleukin-6
INF-γ	Interferon-gamma
LBPs	Live Biotherapeutic Products
LPS	Lipopolysaccharides
MPTP	1-methyl-4-phenyl-1:2,3,6-tetrahydropyridine
NMS	Non-Motor Symptoms
PD	Parkinson’s Disease
QS	Quorum Sensing
ROS	Reactive Oxygen Species
SCFAs	Short-Chain Fatty Acids
SN	Substantia Nigra
SOD	Superoxide Dismutase
TH	Tyrosine Hydroxylase
TNF-α	Tumor Necrosis Factor-alpha
